# Laser Directed Energy Deposition of an AlMgScZr-Alloy in High-Speed Process Regimes

**DOI:** 10.3390/ma15248951

**Published:** 2022-12-14

**Authors:** Tong Zhao, Teng Chen, Yuhan Wang, Mengjie Wang, Maha Bakir, Marius Dahmen, Wangcan Cai, Chen Hong, Thomas Schopphoven, Norbert Pirch, Matthias Brucki, Andres Gasser, Constantin Leon Häfner

**Affiliations:** 1 Fraunhofer ILT—Institute for Laser Technology, Steinbachstraße 15, D-52074 Aachen, Germany; 2 Chair for Laser Technology, RWTH Aachen University LLT, Steinbachstraße 15, D-52074 Aachen, Germany

**Keywords:** aluminum magnesium scandium zirconium alloys, high-speed directed energy deposition—EHLA, cooling rate, track overlapping, porosity

## Abstract

Aluminum-magnesium-scandium-zirconium (AlMgScZr) alloys need to be rapidly cooled from the liquid state to obtain a high degree of solute supersaturation that helps to exploit the precipitation hardening potential of the material. While AlMgScZr alloys have been successfully used in laser powder bed fusion (LPBF) processes, there has been little research in the field of laser directed energy deposition (DED) of the material. The limited previous studies have shown that the performance of AlMgScZr parts fabricated with DED only reached about 60% of that of the parts fabricated with LPBF. In view of breaking through the limitation associated with the process conditions of conventional DED, this work demonstrates the DED of AlMgScZr alloys in high-speed process regimes and elucidates the mechanism of enhancing the hardness and tensile strength of AlMgScZr alloys by increasing the cooling rate by one to two orders of magnitudes, as well as reducing the track overlapping and the porosity of the specimens during the process. A maximum average hardness of nearly 150 HV0.1 and a max. tensile strength of 407 MPa are obtained by using an energy per unit length of 5400 J/m and a powder feed rate per unit length of 0.25 g/m.

## 1. Introduction

5xxx series aluminum alloys (AlMg(Mn)) are lightweight structural materials with an excellent corrosion resistance and weldability. The strength of 5xxx series Al alloys increases with the increase of magnesium (Mg) and manganese (Mn) content; complemented by work hardening, for example, tensile strengths of nearly 400 MPa can be obtained in 5083 (AlMg4.5Mn0.7) Al alloy [1]. Another way to strengthen 5xxx series Al alloys is to supersaturate the material by adding hardly soluble transition elements (TE), such as scandium (Sc), zirconium (Zr), titanium (Ti) etc., allowing the precipitation of fine Al_3_TE dispersoids in solid solution during post-process ageing [2]. Sc is a proven modifier for Al alloys due to its well-studied ability to produce dispersive distributed, L1_2_-structured, and thermal stable Al_3_Sc precipitates that are almost perfectly coherent with the Al matrix [3]. Those secondary Al_3_Sc precipitates which are obtained from the solid Al matrix by ageing and are nano-scale can effectively induce precipitation strengthening and improve the resistance to recrystallisation of the material [4]. When the Sc solute content is greater than that of the eutectic composition (about 0.60 wt% [5] or 0.52 wt% [6]), primary Al_3_Sc precipitates will grow in solidifying liquid and function as heterogenous nuclei which can efficiently refine the microstructure [7]. The nucleation rate of both the primary [8] and secondary precipitates [9] can be further enhanced by supplementing Zr to the alloy. Zr is cheaper and can substitute up to 50% of Sc by forming Al_3_(Sc,Zr) without altering the L1_2_-structure of the phase [10]. The Al_3_(Sc,Zr) phase is thermally more stable than Al_3_Sc because of the lower diffusivity of Zr in Al [11] and a higher configurational entropy (thus is easier to nucleate) of the Al_3_(Sc,Zr) phase [12].

The Mg dissolved in Al raises the lattice constant of the Al matrix [13] and thus reduces the lattice mismatch between the Al matrix and the Al_3_(Sc,Zr) phase, which is already closer to the Al matrix than the Al_3_Sc phase in terms of lattice constant [10]. The critical size of precipitates losing the coherency with the matrix thus increases because of the decreased lattice mismatch [14]. The solid solution strengthening by Mg in Al alloys can be superimposed on the precipitation strengthening by the Al_3_Sc phase [15]. The already low maximum solid solubility (about 0.35 wt% [5] or 0.38 wt% [6]) of Sc (also that of Zr [16]) in Al, however, is further reduced by the presence of Mg [17,18] and limits the precipitation strengthening. The dissolved amount of Sc cannot be increased simply through adding more Sc to the alloy, since the atom will be directly consumed in primary precipitates [19] or, if the solidification is slow or the material is kept at high temperatures (about 450 °C), in particles formed by discontinuous precipitation [14].

An alternative way to trap more Sc and Zr in AlMg alloys is to increase the cooling rate during the solidification. Early studies done under laboratory conditions demonstrate that small-sized cast parts can have a Sc content of up to 0.5–0.6 wt%, which is anomalously higher than that of large ingots produced under shop conditions [20]. More precise measurements show a significant improvement of both the as-cast hardness (about 30 HV10 vs. 43 HV10) and the peak hardness increment after ageing (about Δ12 HV10 vs. Δ29 HV10) when the cooling rate is increased from 0.16 K/s to 7.69 K/s in casting of an Al-0.3Sc-0.15Zr (wt%) in alloy [21]. In cast parts with the composition of Al-4Mg-0.4Sc-0.12Zr (wt%), electron beam remelting of the part surface (cooling rates up to 10^5^ K/s) shows the possibility to get 0.37 wt% Sc (vs. 0.12 wt% in as-cast state) into the solid solution and thus higher hardness after ageing [22]. Using melt spinning, where thin ribbons (20–100 µm) are cast and cooling rates reach up to 10^5^ K/s–10^7^ K/s, at least 1.33 wt% Sc and 1.84 wt% Zr can be forced into the solid solution [23]. A high degree of supersaturation satisfies the prerequisite for obtaining a high volume-fraction of secondary precipitates. Also decisive for strengthening the material is the precipitate size, which can be fine-tuned by ageing [23,24,25].

Over the past three decades, laser powder bed fusion (LPBF, also termed SLM) [26] has gained tremendous popularity in metallic additive manufacturing. Materials (initially as powder particles) in LPBF are rapidly melted and solidified in a way that resembles electron beam processing and melt spinning. The earliest study on LPBF of AlMgScZr alloys has demonstrated the feasibility of achieving nearly 500 MPa tensile strength, 475 MPa yield strength, and 160 HV0.1 hardness (after 4 h ageing at 300 °C) [27]. Since then, many studies have emerged, representative of which focus on process parameter optimization [28], microstructure characterization [29], post-process heat treatment [30], customization of material composition [31], etc. High cooling rates (as high as 10^6^ K/s [32]) in LPBF are considered to meet the process requirement for AlMgScZr alloys well [27]. Increasing the scanning speed has led to an increase in the Sc/Zr content in the Al matrix [33], as well as the hardness of the material after ageing [27].

Another technology that is functionally complementary to LPBF and more suitable for large-size additive manufacturing, cladding, or repairing of existing parts is laser beam directed energy deposition (hereinafter referred to as DED, also termed LMD in the literature), where powders or wires are fed by nozzles and deposited by laser melting [34]. Compared to LPBF, the laser spot used in DED is usually larger, and the travel (or scanning) speed is orders of magnitude lower [35]. Cooling rates in DED normally vary from approx. 10^2^ K/s to 10^4^ K/s depending on the selected parameters and the material to be processed [36,37,38]. Comparative studies of the two processes of AlMgScZr alloys have shown that the strength obtained in DED is only about 60% of that in LPBF [39]. Critical cooling rates for suppressing the nucleation of primary precipitates of an Al-3.3Mg-0.68Mn-xSc-yZr alloy are roughly estimated at 110 K/s for a low (0.25Sc-0.12Zr) and at 3 × 10^4^ K/s for a high (0.50Sc-0.21Zr) Sc/Zr content. Since 3 × 10^4^ K/s is already near the aforementioned upper limit, a hardness of only 105 HV0.2 (after 4 h ageing at 325 °C) with a very weak ageing response (about Δ10 HV0.2) is obtained in DED under normal process settings even with the high Sc/Zr content [40]. The 3D-parts in DED are built up by stacking of layers which are formed by a lateral overlap of deposition tracks. Preliminary studies on ageing response of DED tracks of AlMgScZr alloys show a nonuniform hardness distribution with soft fringes and hard cores on track cross-sections [41]. The layer stacking and track overlapping results in the harder core of previous tracks being covered by the softer fringe of following tracks, leading to a low overall hardness level at high overlap rates [42].

**Figure 1:**
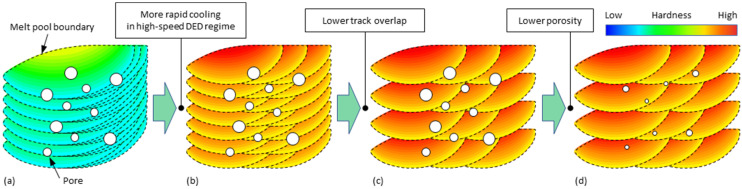
Approach to improve the mechanical performance of AlMgScZr alloys in DED as illustrated by the hardness distribution over the sample cross-section obtained by overlapping tracks deposited under different conditions: (**a**) conventional DED, (**b**) high-speed DED with high track overlap rate, (**c**) high-speed DED with decreased track overlap rate, (**d**) with further decreased porosity.

In view of breaking through the limitation associated with the cooling condition of DED, it is considered necessary to expand the scope of research by increasing the travel speed from the conventional (about 1 m/min) to high-speed (about 20 m/min or more) regime. While enhancing the strengthening potential of individual tracks in this way, more harder track cores need to be preserved to improve the multi-layer performance overall. DED parts have been successfully built with scan speeds up to 40 m/min, but defects like high porosity still point to room for improvement [43]. By addressing these issues in the experiment, this work studies, in more detail, methods to improve the reachable hardness and strength of AlMgScZr alloys in DED, as shown in the approach in Figure 1.

## 2. Materials and Methods

### 2.1. Powder

**Figure 2:**
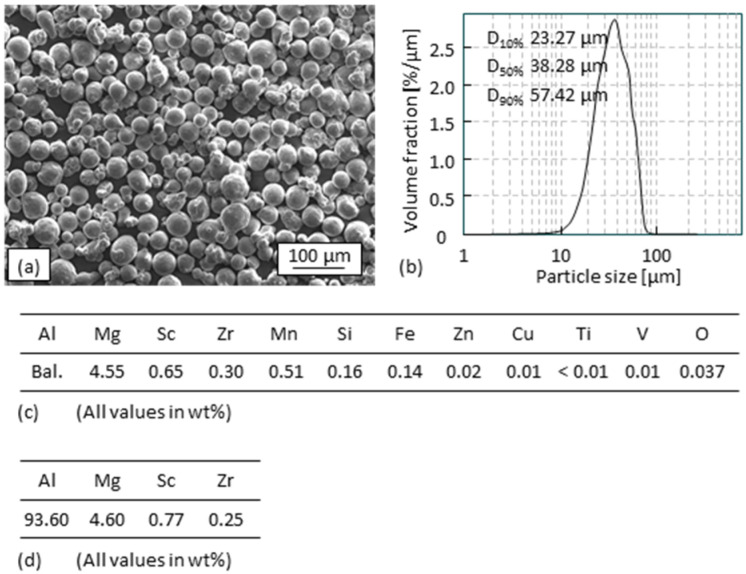
AlMgScZr alloy powder used in this work: (**a**) SEM image, (**b**) powder particle size distribution, (**c**) composition of the powder according to the data sheet, (**d**) content of Al, Mg, Sc, and Zr checked with ICP-OES.

A commercially available gas-atomized AlMgScZr alloy powder with the composition of Al-4.55Mg-0.65Sc-0.30Zr (wt%) was used in the experiments. A SEM (scanning electron microscopy) (LEO 1455 EP of Carl Zeiss AG, Oberkochen, Germany) image of the powder is shown in Figure 2a. The particle size fraction of the powder with D_10%_ = 23.27 µm, D_50%_ = 38.28 µm, D_90%_ = 57.42 µm is shown in Figure 2b. The chemical composition of the powder according to the data sheet offered by the supplier is shown in Figure 2c. The content of Al and of the main alloy element Mg, Sc, and Zr checked by ICP-OES (SPECTROBLUE of SPECTRO Analytical Instruments GmbH, Kleve, Germany) (inductively coupled plasma optical emission spectroscopy) of a small amount sampled from the powder are shown in Figure 2d. The composition data are consistent with each other.

### 2.2. Experimental Setup

Two types of specimens, namely single tracks and hollow-cylindrical shaped 3D-parts were produced. The single tracks were fabricated in large quantities to identify the influence of variation of main process parameters (laser power *P*, travel speed *v*, powder feed rate *ṁ*) across conventional and high-speed process regimes on the ratio of material deposition to melt pool dilution by measuring the geometry of the cross-sections of the tracks (more details see Section 2.5). Representative parameters were selected and then used to build 3D-parts.

**Figure 3:**
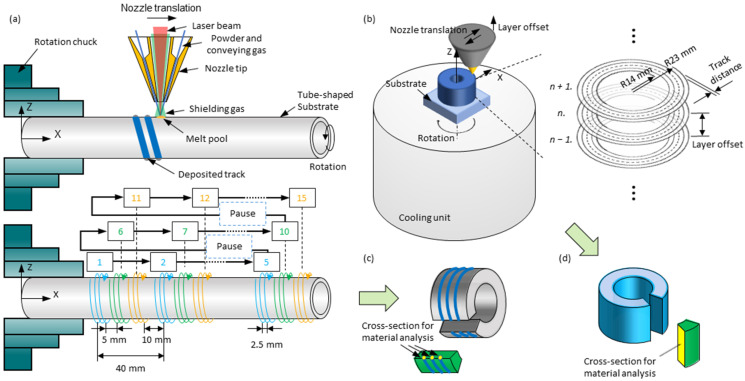
Schematic illustrations of the process strategy for fabricating (**a**) single tracks and (**b**) 3D-parts, and of their respective (**c**,**d**) sampling approaches.

For fabricating single tracks, 5083 alloy tubes were used as substrates (250 mm length, 33.4 mm outer diameter, 3.38 mm wall thickness, at room temperature) that were clamped on a chuck and rotated with the spindle, as shown in Figure 3a (above). The powder nozzle moved in the direction parallel to the spindle axis and with 10 mm (i.e., nozzle distance) stand-off distance to the substrate surface. Powder particles were conveyed by Ar (i.e., conveying gas) in a conical beam to the substrate surface and melted by a coaxially aligned laser beam, along which there was an additional Ar flow (i.e., shielding gas) for enhancing the shielding performance. Materials were deposited in a melt pool moving with the travel speed relative to the substrate. Maximum 15 helical tracks could be fabricated on one tube-shaped substrate. The tracks were spaced to prevent mutual thermal influences, as shown in Figure 3a (below). For fabricating the 3D-parts, every time, a piece of substrate (5083 Al alloy, 12 mm thickness) was fixed in the center of the end face of a cylindrical aluminum block (5083 Al alloy, pre-cooled by ice water to 0 °C, Ø 200 mm, 200 mm height) which rotated along an axis parallel to the powder nozzle axis, as shown in Figure 3b. The powder nozzle translated in the radial direction (inner diameter = 28 mm, outer diameter = 44 mm) of the rotation back and forth. The laser was on while the nozzle was moving from the inner to the outer radius. The rotation speed was automatically adjusted to the radial position of the nozzle to ensure a constant linear speed (i.e., the travel speed) between the nozzle and the substrate surface. As a result, spiral tracks that build layers were formed. The distance between the nozzle tip and the growing specimen was adjusted after each layer was formed. For material analysis (Section 2.5) on cross-sections, small pieces of samples were cut from the specimens, as shown in Figure 3c,d.

**Figure 4:**
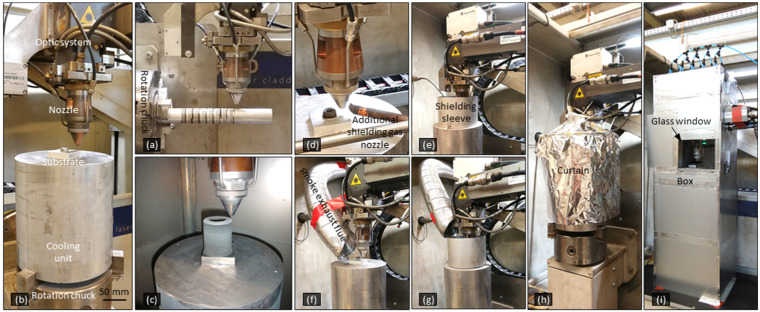
Photos of the basic experimental setup for fabricating (**a**) single tracks and (**b**) 3D-parts “Or”. Most of the 3D-parts like that shown in (**c**) were made with additional shielding concepts: (**d**) an additional shielding gas flow “Ad”, (**e**) a shielding sleeve “Ri”, (**f**) a smoke exhaust flue “Ex”, (**g**) a combination of the sleeve and the flue “RE”, (**h**) an Al curtain “Fo”, and (**i**) a protective box “Bo”. The designators in quotation marks are the short forms used in Table 1. “Bo” has a larger interior space and is thus more heat resistant than “Fo“. The letters in quotation marks are specimen designations (see Table 1).

**Figure 5:**
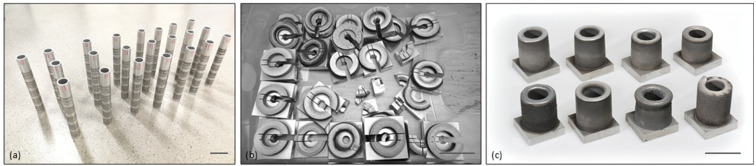
Exterior views of specimens of (**a**) single tracks, (**b**) 3D-parts with about 5 mm height and used for hardness tests, and (**c**) 3D-parts with about 50 mm height and used for tensile tests. The black bars indicate a length of 50 mm.

The experiment was carried out in an EHLA (acronym of extreme high-speed laser metal deposition) system, which integrated a lathe-like CNC machine (max. spindle speed 775 rpm, Hornet Laser Cladding BV) with the laser cladding optics (laser spot Ø 1.3 mm, BEO D70, Trumpf GmbH + Co. KG) and powder-feeding assembly (Nozzle of Fraunhofer.ILT, Germany; powder feeder TWIN-150 of Oerlikon Mecto AG, Switzerland). High travel speed was achieved by combining the nozzle translation and the high-speed spindle rotation. The spindle axle is perpendicular or parallel to the nozzle axis, thus creating the single tracks or 3D-parts, respectively, as shown in Figure 4a–c. Since sufficient shielding gas flow is observed to improve the build quality of 3D-parts in DED [43], and considering that the nozzle’s own shielding gas flow can be inadequate under open atmosphere when the travel speed is high, several shielding concepts were compared. For this, an additional shielding gas flow, a shielding sleeve, a smoke exhaust flue, an Al curtain, and a protective box were used, as shown in Figure 4d–i. It should be noted that because of the difficulty of sealing due to the spindle rotation, the process was done out of chamber, and none of the methods could guarantee an absolute inert atmosphere around the meld pool. Some examples of the specimens are shown in Figure 5.

### 2.3. Process Variables

During the DED process, a millimeter-sized melt pool moves along the relative motion trajectory between the powder nozzle and the substrate. Under the simplifying assumptions that (a) the material physical properties are independent of temperature, (b) travel speed and heat input are constant, (c) a point heat source, (d) the heat flow around the heat source is in quasi-stationary state, (e) the substrate is thick and can be treated as semi-infinite, (f) no phase transition enthalpy, and (g) the surface heat losses can be ignored, the cooling rate [K/s] at the solidification front on the central line of the moving melt pool is given by Rosenthal’s equation [44] as follows:

(1) $${\left.\frac{dT}{dt}\right|}_{T=T_{l}}=-2\pi \cdot k\cdot \frac{1}{\alpha }\cdot \frac{v}{P}\cdot {\left(T_{l}-T_{0}\right)}^{2}$$

where *k* is the material thermal conductivity [W/(m∙K)], *α* is the absorption rate [1], *v* is the travel speed [m/s], *P* is the laser power [W], *T_l_* is the liquidus temperature [K], and *T*_0_ is the initial temperature of the solid part [K].

For a given material, the cooling rate at the solidification front can be increased by decreasing *T*_0_ or increasing *v*/*P*. The term

(2) $$\frac{P}{v}=E^{\prime }$$

is also known as *E’* or energy per unit length [J/m]. The cooling rate in different process regimes can be roughly estimated by inserting the values *k* = 120 W/(m∙K) (at 20 °C) and *T_l_* = 875.15 K (638 °C) for the 5083 Al alloy [45], *α* = 0.24 for the 5251 Al alloy (AlMg2Mn0.3, surface roughness S_a_ = 0.28 µm, at 20 °C, 1053 nm wavelength) [46], and the different *T*_0_, *v*, and *P* into Equation (1). For the investigated values of high-speed DED, e.g., *T*_0_ = 273.15 K (0 °C), *v* = 0.33 m/s (20 m/min), and *P* = 1800 W, the cooling rate is estimated to 2.34 × 10^5^ K/s, which is much higher than that in conventional DED with the values of, e.g., *T*_0_ = 293.15 K (20 °C), *v* = 0.0083 m/s (0.5 m/min), and *P* = 600 W, with an estimated cooling rate of 1.67 × 10^4^ K/s, or *T*_0_ = 373.15 K (100 °C), *v* = 0.015 m/s (0.9 m/min), and *P* = 1800 W, with an estmated cooling rate of only 7.58 × 10^3^ K/s. As a result of this estimation, the cooling rates in the high-speed process regime are one to two orders of magnitude higher than in conventional DED.

**Figure 6:**
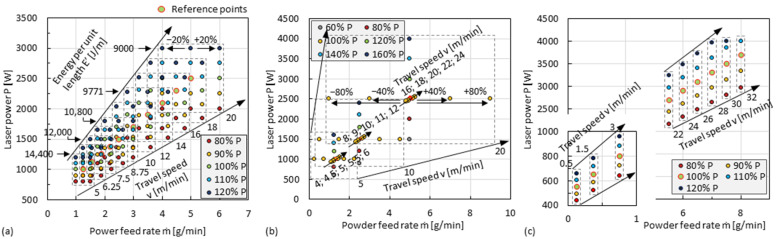
Parameter sets of laser power *P*, travel speed *v*, and powder feed rate *ṁ* for fabricated single tracks. (**a**) The reference points are set according to *P* [W] = 500 [W] + 100 [W∙min/m] × *v* [m/min] and *ṁ* [g/min] = 0.25 [g/m] × *v* [m/min], and the extended points are obtained by varying *P* (±10%, ±20%) and *ṁ* (±20%) proportionally, and the travel speed of each of the 15 points framed by a dotted line is the same; (**b**) Further points are obtained by varying *P* (60–160%), *ṁ* (±40%, ±80%), and *v* (±10%, ±20%) proportionally based on the reference points of (**a**) at *v* = 5 m/min, 10 m/min, and 20 m/min; (**c**) Further reference points are set according to the same equations of (**a**), and *v* = 22~32 m/min, and the extended points are obtained by varying the *P* (±10%, ±20%) proportionally, and the max. *P* is 4000 W. Laser spot Ø = 1.3 mm. Conveying gas flow rate = 5 l/min. Shielding gas flow rate = 10 l/min.

It is important to note that the ideal parameter values for increasing the cooling rate must be able to guarantee the real material deposition. To figure out the feasible maximum of *v*/*P* and considering that the powder feed rate *ṁ* should have influence on the track overlapping, process parameters for fabricating single tracks were selected in a large range of settings that covers *P* from 800 W to 4000 W, *v* from 5 m/min to 32 m/min, *ṁ* from 0.125 g/min to 8 g/min, as shown in Figure 6. Values of *E’* = *P*/*v* of some parameter sets are given in Figure 6a. Another value, powder feed rate per unit length [g/m], which is defined as *m’* = *ṁ/v*, will be used later in the Results Section. Representative values of the parameters were then selected for fabricating 3D-parts, as shown in Table 1. For the specimens Or1 and Ad1–Ad5, material deposition occurred when the nozzle moved in both directions between the inner and outer diameter of the specimen; for the other specimens, the laser was switched on, and thus the material deposition occurred only when the nozzle moved from the inner diameter to the outer diameter.

### 2.4. Heat Treatment

An optimum ageing heat treatment should be addressed in AlMgScZr alloys to control the precipitation hardening to acquire the desired high-performance mechanical properties. Ageing at low temperatures of about 300 °C is suggested to allow the formation of coherent and homogeneously distributed Al_3_Sc precipitates in Al-0.2Sc (wt%) alloy [14]. Casted Al-0.41Sc (wt%) alloy is found to be peak-age hardened by heating the material at temperatures between 300 °C and 350 °C for about 3 h [47]. As for AlMgSc(Zr) alloys, results of previous studies on optimum heat treatment strategies leading to the highest tensile strength or hardness are shown in Table 2. To study the process-related ageing response (i.e., the precipitation hardening potential), specimens in this work were sampled, as shown in Figure 3d, and aged at 300 °C, 325 °C, or 350 °C for 3, 4, 5, 7, or 9 h and then air-cooled for comparison. Considering the result and for ease of comparison with previous studies, “4 h at 325 °C + air cooling” was then selected and used to treat the specimens to study the influence of other factors like porosity and layer overlap ratio on the hardness.

Supersaturated Mg in Al alloys can precipitate forming the phase transforming from Guinier-Preston zones, to β’’ phase, then to β’ phase and finally to β phase [50]. The β’ (Al_3_Mg_2_) phase is semi-coherent and found to be the main hardening phase. The formation of β’ phase starts, according to the study on Al-8Mg and Al-12Mg (wt%) alloy, already at 100 °C and can be accelerated (i.e., tens of hours) at 150 °C [51]. Since Mg in Al-4.55Mg (wt%) (Al-5Mg at%) alloy will dissolve into the matrix at above 235 °C [52], which is much lower than the 325 °C ageing temperature (for Sc/Zr precipitation), Mg cannot be fully utilized by a single one-stage ageing. Therefore, double-stage ageing strategies were also attempted. Specimens were firstly aged at 325 °C for 4 h, then water-cooled, and finally aged at 150 °C for 1–4 h, or at 70 °C for 2–30 h.

### 2.5. Material Analysis

**Figure 7:**
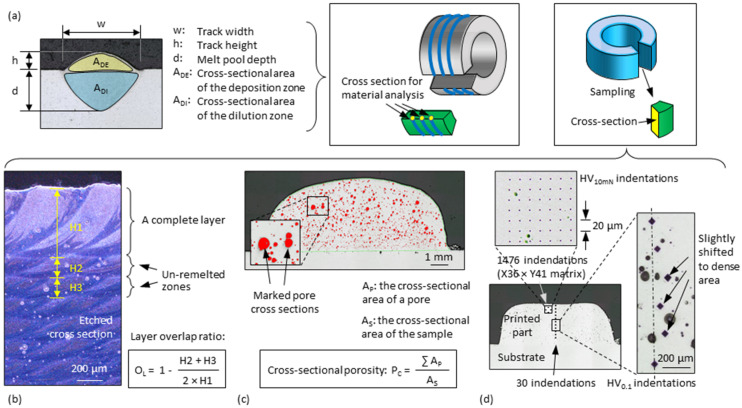
Schematic illustrations of the different material analyses: (**a**) dimension measurement of the cross-section of a single track (indicated by the blue lines); (**b**) measurement of layer overlap ratio of a 3D-part; (**c**) measurement of the cross-sectional porosity of a 3D-part; (**d**) hardness measurement by micro- and nanoindentation of a 3D-part.

Specimens of single tracks and 3D-parts were cut as shown in Figure 3c,d, allowing the analysis on track cross-sections. The specimens were cold embedded. The cross-sections were wet ground until using 1200 grid SiC sandpaper and then polished (18 N for 20 min) with 1 µm diamond suspension. The polished cross-sections of the single tracks were additionally etched with a sodium hydroxide solution (NaOH, 10%). The microstructural details were thus visible on the microscope and dimensions like track width *w*, track height *h*, melt pool depth *d*, and the cross-sectional area of the deposition zone *A_DE_* (i.e., the melting part above the substrate surface) and of the dilution zone *A_DI_* (i.e., the melting part below the substrate surface), as shown in Figure 7a. Some samples cut from the 3D-parts were also etched so that the thickness of the layers formed by overlapped tracks could be measured, as shown in Figure 7b. *H2* represents the remaining visible height of the second last layer when it is partially remelted by the last layer. *H3* represents the remaining visible height of the third last layer when it is partially remelted by the second last layer. The overlap between the last and the second last layer is thus 1 − (*H2*/*H1*), and the overlap between the second last and the third last layer is 1 − (*H3*/*H1*) (assuming a constant layer height of *H1*). The average of the two values is defined in this work as the layer overlap ratio *O_L_*. With the help of contrast difference due to illumination, cross-sectional porosities *P_C_* were measured, as shown in Figure 7c. The porosity was defined as the ratio between the sum of the dark pore areas to the total cross-sectional area of the specimen. Microhardness measurements were carried out according to Vickers with the test load of 100 g (HV0.1). One measurement consisted of 20–30 indentations with a point spacing of approx. 0.2 mm centrally along the specimen. The indentation positions were created as one line and then slightly shifted to the dense zone when the points encountered pores, as shown in Figure 7d (right). A pico/nanoindenter was used to draw hardness mapping diagrams of the last several layers of some specimens. Each measurement was performed in a 36 × 41 (700 μm × 800 μm) matrix with a total of 1476 indentations, as shown in Figure 7d (above). In addition, tensile tests were conducted (DIN EN ISO 6892-1:2017-02, room temperature) by using specimens (DIN 50125:2009–07, B4 × 20) taken from the full-height (about 50 mm) 3D-parts, as shown in Figure 5c.

## 3. Results

### 3.1. Dilution Zone of Single Tracks

**Figure 8:**
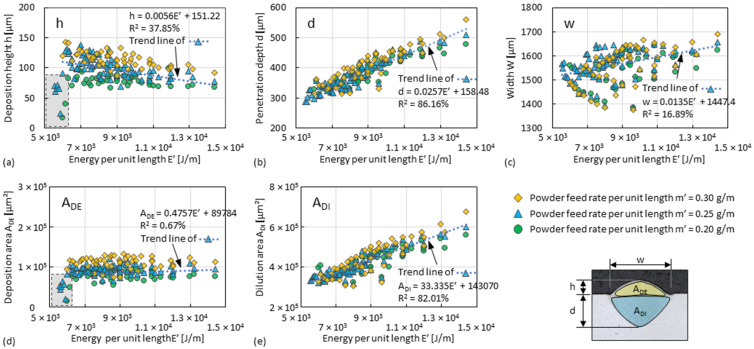
Plot graphs of (**a**) track height *h*, (**b**) melt pool depth *d*, (**c**) track width *w*, (**d**) cross-sectional area of the deposition zone *A_DE_*, and (**e**) cross-sectional area of the dilution zone *A_DI_* against energy per unit length *E’* with different powder feed rates per unit length *m’*. Every point refers to the average value of three measurements. R^2^ refers to coefficient of determination for the trend line.

Results of the dimension measurements (i.e., *h*, *d*, *w*, *A_DE_*, and *A_DI_*, according to Figure 7a) of the single tracks fabricated with different sets of P, v, and ṁ (according to Figure 6) are plotted against *E’*, as shown in Figure 8 (plot directly against *P* is shown in in Appendix A). It is apparent from the diagrams that *h* and A_DE_, which refer to the size of the deposition zones, are not much influenced by *E’*, but slightly increase with *m’*, as shown in Figure 8a,d. When *E’* increases, w exhibits only a weak tendency to increase, as shown in Figure 8c. Significant proportional are *d* and *E’*, as well as *A_DI_* and *E’*, as shown in Figure 8b,e. This indicates that the increase in *A_DI_* (since *A_DI_*~*d* × *w*) caused by the increase in *E’* is more related to the increase in *d*. In the low values *E’* region and thus low energy input, as marked by the dashed box in Figure 8a,d, *h* and *A_DE_* decrease faster, implying a reduction in material catch rate. Although a lower *E’* value (thus a higher *v/P*) leading to higher cooling rates is desired, the minimum threshold of *E’* to ensure the material deposition at current process settings in single tracks is about 5500 J/m (corresponding to *d* ≈ 300 µm, *A_DI_* ≈ 3.26 × 10^5^ µm^2^).

**Figure 9:**
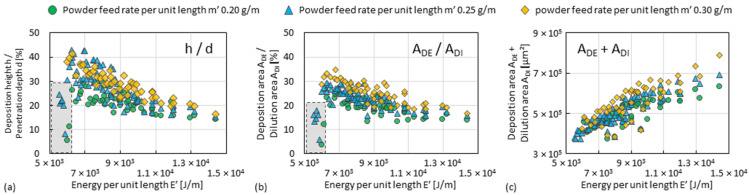
Plot graphs of the index (**a**) ratio of *h*/*d*, (**b**) ratio of *A_DE_*/*A_DI_*, and (**c**) sum of *A_DE_* + *A_DI_* against *E’*. Every point refers to the average value of three measurements.

Important indices that control the overlapping of adjacent tracks within a layer or between layers are the ratio of *h*/*d* and of *A_DE_*/*A_DI_*. A higher ratio of *h*/*d* or of *A_DE_*/*A_DI_* indicate that less of the deposited material (i.e., that of last track or layer) will be remelted relative to the material being deposited. The two indices increase as *E’* decreases until it reaches the threshold of about 6000 J/m. They can be increased to about 40% or 32%, respectively, by the increase in *m’* (here, to 0.30 g/m), as shown in Figure 9a,b. The sum of *A_DE_* and *A_DI_* decreases as *E’* decreases, as shown in Figure 9c, which means that the total heat input is reduced.

### 3.2. Deposition Zone of Single Tracks

**Figure 10:**
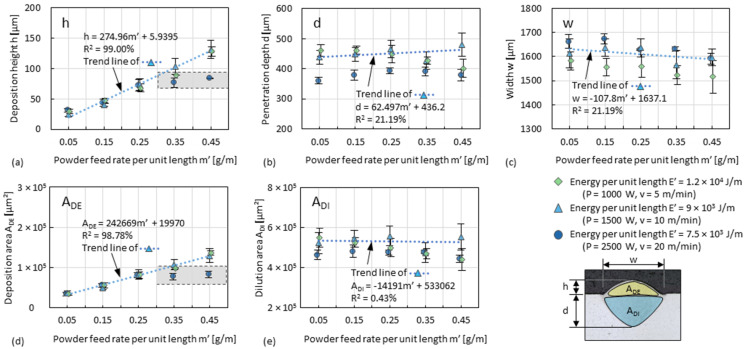
Plot graphs of (**a**) track height *h*, (**b**) melt pool depth *d*, (**c**) track width *w*, (**d**) cross-sectional area of the deposition zone *A_DE_*, and (**e**) cross-sectional area of the dilution zone *A_DI_* against powder feed rate per unit length *m’* with different energy per unit length *E’*. Every point refers to the average value of three measurements. *R^2^* refers to coefficient of determination for the trend line.

**Figure 11:**
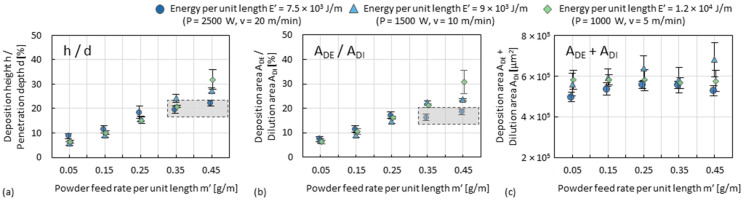
Plot graphs of the index (**a**) ratio of *h*/*d*, (**b**) ratio *A_DE_*/*A_DI_*, and (**c**) sum *A_DE_* + *A_DI_* against *m’*. Every point refers to the average value of three measurements.

The influence of powder feed rate and travel speed on the dimensions (i.e., *h*, *d*, *w*, *A_DE_*, and *A_DI_*, according to Figure 7a) of single tracks (fabricated according to Figure 6b) are more clearly seen by plotting the data against *m’*, as shown in Figure 10. The influence of *m’* and *E’* on *d*, *A_DI_* and *w*, as shown in Figure 8, is also depicted in Figure 10b,c,e. *h* and *A_DE_* strongly increase as a function of *m’*, whereas *E’* has only a minor influence, as shown in Figure 10a,d. This indicates that the deposition zone becomes slender (since *A_DE_* ~ *w* × *h*) as *m’* increases. At a value of *E’* of 7500 J/m (i.e., the lowest in the diagrams), both *h* and *A_DE_* reach a plateau of about 80 µm and 8 × 10^4^ µm^2^, respectively, as *m’* increases between 0.25 g/min and 0.35 g/min. This plateau indicates that while decreasing *E’* to obtain high cooling rates and small dilution zones, increasing *m’* to obtain large deposition zones is limited by an insufficient melting of powder. The plateau can also be seen in the diagram of the ratio of *h*/*d* and of *A_DE_*/*A_DI_*, as shown in Figure 11a,b. Although the trend of the sum of *A_DE_* + *A_DI_* as a function of *m’* is nearly horizontal, as shown in Figure 11c, *d* or *A_DI_* cannot be arbitrarily increased just by increasing *m’*.

**Figure 12:**
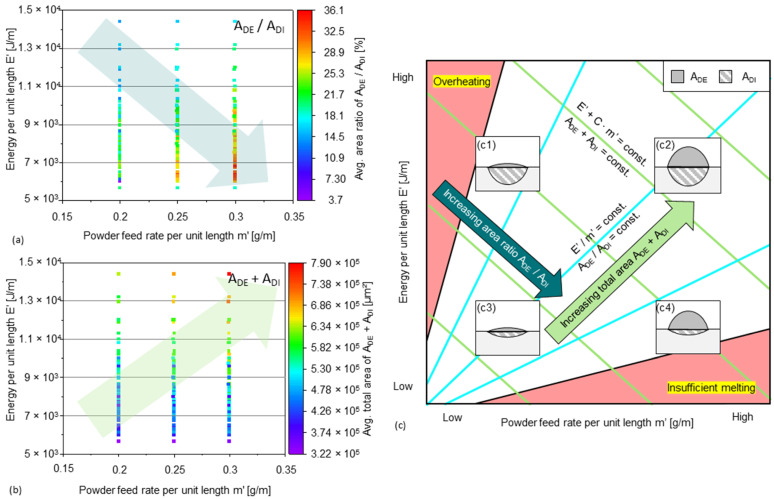
The influence of *E’* and *m’* on (**a**) *A_DE_*/*A_DI_* and (**b**) *A_DE_* + *A_DI_* represented by real data and by (**c**) a schematic diagram where the subfigures represent four different area ratios.

A summary of the data of the influence of *E’* and *m’* on *A_DE_*/*A_DI_* and *A_DE_* + *A_DI_* is shown in Figure 12a,b. The influence can be represented schematically in the diagram shown in Figure 12c. As *E’* increases, more energy is transferred into the dilution part. With increasing m’, higher tracks are realized, and more energy is absorbed by the deposition part. A low *E’* allows a high cooling rate and a high *m’* allows a high deposition rate. However, ideal tracks that enable high-rate deposition having little heat effect on previous layers and are cooled quickly (i.e., type c4) are difficult to fabricate due to limitations like insufficient melting or possible defective binding. A feasible combination of a low *E’* (e.g., 6000 J/m) and a relative high *m’* (e.g., 0.25 g/m) can be found that leads to an *A_DE_*/*A_DI_* ratio of about 30% and a sum of *A_DE_* + *A_DI_* of about 4 × 10^5^ µm^2^, as shown in Figure 12a,b. During the fabrication of 3D-parts, the conditions change dynamically as the substrate temperature rises or the heat dissipation conditions change. Nevertheless, a qualitative approach to choose proper parameter values is given by the diagrams, as well as the understanding of how the geometry of single tracks is influenced by the parameters.

### 3.3. Ageing of 3D-Parts

**Figure 13:**
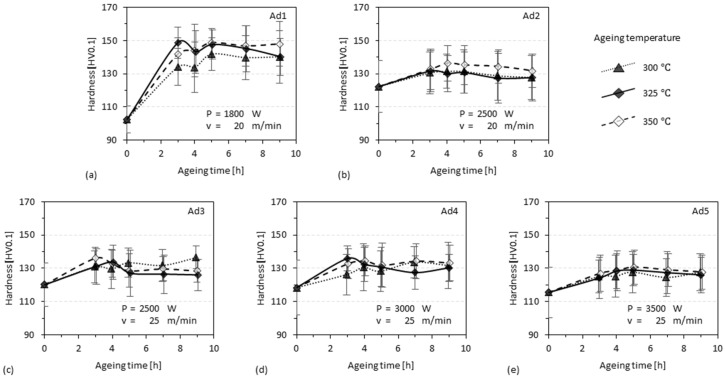
Microhardness profiles over ageing time of the specimens sampled from (**a**) Ad1, (**b**) Ad2, (**c**) Ad3, (**d**) Ad4, and (**e**) Ad5. The aging time 0 h stands for the as-built state. *P*: laser power [W], *v*: travel speed [m/min]. Every value corresponds to the mean value of 30 indentations. Error bar: 1 *σ*.

The values of parameter *P* and *v* have been chosen so that the *E’ (=P/v)* is within the low value range according to Figure 12. The powder feed rate was set to *ṁ* = 5 g/min so that *m’* (0.20 g/m, 0.25 g/min) was in the zone with a relative high *A_DE_*/*A_DI_* and low *A_DE_* + *A_DI_*. The hardness profiles of five specimens fabricated under the same conditions by varying only the laser power *P* and travel speed *v* are shown in Figure 13. The profiles exhibit hardness data of the specimens in an as-built state and of the specimens that were aged at 300 °C, 325 °C, or 350 °C for 3 to 9 h. It is noticeable that Ad1, whose *E’* is the lowest (i.e., 5400 J/m), has the lowest hardness in the as-built state (102 HV0.1) but obviously the highest ageing response (max. 149 HV0.1, Δ 47 HV0.1, 5 h at 325 °C), as shown in Figure 13a. The other specimens also have a relative high level of hardness (in comparison with that in Table 2), but the peak hardness (about 130 HV0.1) is lower and the ageing response (about Δ15 HV0.1) is weaker than that of Ad1, as shown in Figure 13b–e. One more thing learnt is that the hardness plateaus for all specimens were obtained after 4 h at the latest. A longer aging time up to 9 h could not produce a further increase in hardness.

### 3.4. Cross-Sectional Porosity of 3D-Parts

**Figure 14:**
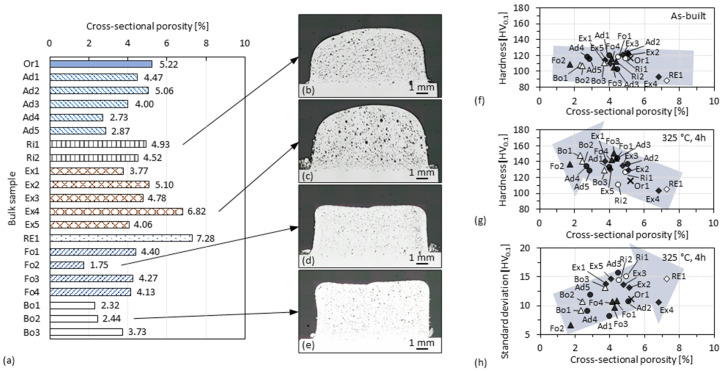
(**a**) Cross-sectional porosity of the specimens fabricated with shielding and exhausting measures; (**b**–**e**) some representative cross-sections of the specimens; plot graphs of the hardness against the cross-sectional porosity in the (**f**) as-built state or (**g**) aged state, and of (**h**) the standard deviation of the hardness values against the cross-sectional porosity. Each hardness data point represents the average of 30 indentations.

Cross-sections of the specimens listed in Table 1 were sampled and prepared for comparing their cross-sectional porosity with each other, as shown in Figure 14a. Except for the specimens Ad1–Ad5, other specimens were uniformly fabricated with a travel speed *v* of 20 m/min and a lower level of laser power *P* (i.e., 1800 W–2500 W). Although overall porosity of each sample is still high (i.e., up to about 5%), the difference in the effect of shielding and exhausting measures on porosity can be seen. The active exhaust was used to prevent more soot from settling on the specimen surface being cladded. The porosities of the specimens (i.e., Ex1–5 and Re1) fabricated with the exhaust, however, are apparently higher, perhaps because the local shielding atmosphere was destroyed by the exhaust airflow. Specimens fabricated with the shielding sleeve (i.e., Ri1–2 and Ex1–5) have a more rounded cross-sectional profile in addition to a higher porosity, as shown in Figure 14b–e. Although the same cooling unit was used, the narrow shielding sleeve may have induced a stronger laser reflection onto the specimen and therefore caused these specimens to be overheated (thus lower cooling rates) in the process. Other shielding measures with more spacious conditions have been observed to allow the fabrication of samples (i.e., Ad4–5, Fo2 and Bo1–2) having significantly lower porosity (i.e., about 2%) than the reference specimen fabricated with the original setting (i.e., Or1). However, specimens with about 4–5% porosity were also observed, indicating that the shielding measures alone were still insufficient to ensure the fabrication of fully dense specimens (further discussion in Section 3.7.

Nevertheless, the benefits of reducing porosity for increasing hardness can be seen by plotting hardness versus porosity of the specimens, as shown in Figure 14f–g. Hardness indentations were placed so that each individual value was measured in an area that was dense in the cross section but might still be influenced by surrounding pores. Since each specimen was fabricated with different thermal conditions, a direct comparison between the as-built hardness is not necessarily meaningful. However, as mentioned in Section 3.3 hardness plateaus that eliminate the influence of the previous thermal history can be reached after 4 h ageing. In the aged specimens, an increase in hardness with a decrease in porosity can be clearly observed. The standard deviation of the hardness values measured on each specimen also varies with porosity with a roughly increasing trend. These data points are distributed in a discrete manner, indicating that other factors besides porosity can affect hardness.

### 3.5. Layer Overlap Ratio of 3D-Parts

**Figure 15:**
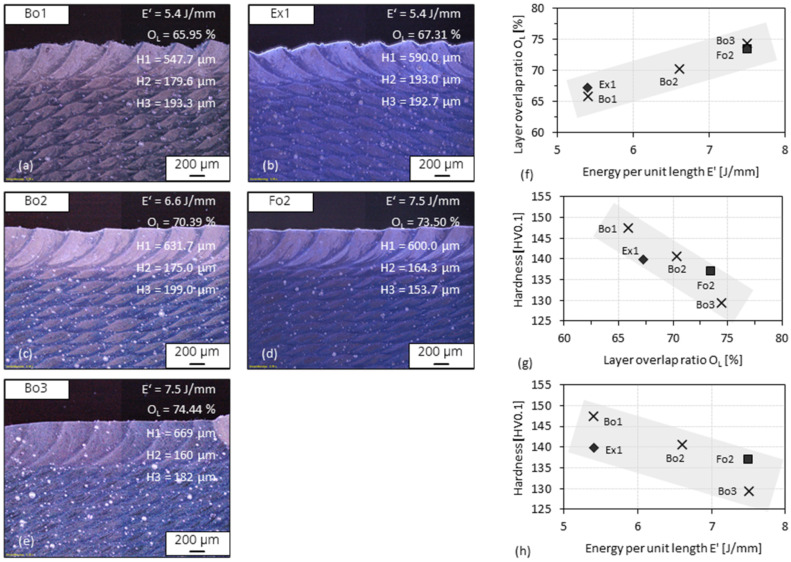
Microscopic dark field images of the etched specimen cross-section of (**a**) Bo1, (**b**) Ex1, (**c**) Bo2, (**d**) Fo2, and (**e**) Bo3. (**f**) Plot graph of layer overlap ratio O_L_ against energy per unit length E’. Plot graphs of hardness against layer overlap ratio O_L_ (**g**) and energy per unit length E’ (**h**). The thickness H1, H2, and H3 marked in (**a**–**e**) are each the average value of three measurements. Each hardness data point represents the average of 30 indentations. The specimens had been aged at 325 °C for 4 h.

Specimens which have relatively low porosities and were fabricated with a stepwise variation of the energy per unit length *E’* (=*P*/*v*) as well as the same travel speed and scan strategy were selected. Layer thickness was measured on etched specimen cross-sections to calculate the layer overlap ratio *O_L_* (defined in Figure 7b), as shown in Figure 15a–e. The relationships between *E’* and *O_L_* as well as the hardness (after ageing at 325 °C for 4 h) are shown in Figure 15f–h. The relationship between the calculated *O_L_* and the corresponding *E’* is shown in Figure 15f. A lower *O_L_* or *E’* correlates with a higher hardness level, as shown in Figure 15g–h, so that the average hardness value of Bo1 (147 HV0.1) is about 17 HV0.1 higher than that of Bo3. The deviation of the hardness values is consistent with that observed in Figure 14 regarding the porosity.

**Figure 16:**
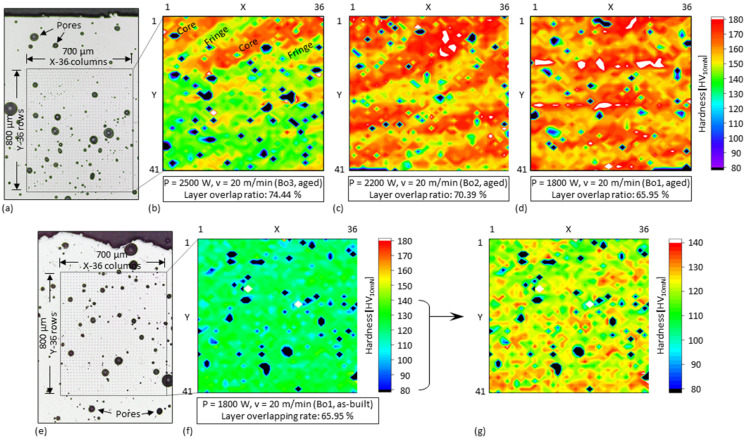
Microscopic images of the cross-section of the top layers of the specimen (**a**) Bo3 and (**e**) Bo1. Color-coded contour maps of the hardness distribution of aged (325 °C for 4 h) (**b**) Bo3, (**c**) Bo2, and (**d**) Bo1 and of (**f**,**g**) as-built Bo1. The (**b**–**d**) and (**f**) have the same color scale, which is partially enlarged in (**g**).

To understand the effect of *O_L_* and *E’* on the hardness, hardness mapping was performed in matrices covering several track cross-sections each in the top (i.e., the last deposited) layers of the specimen Bo1, Bo2, and Bo3, as shown in Figure 16. The process parameters of the specimens differ only in the laser power. The test load used was 10 mN (i.e., ~1/100 of HV0.1), allowing densely arranged (20 µm distance) µm-sized indentations as shown in Figure 16, so that the local hardness of the material among pores can be precisely measured without the interference of pores like that observed in microhardness tests (Figure 14 and Figure 15). The material at the boundaries of the tracks after ageing are generally softer (about 130–150 HV_10 mN_) than in the cores (about 160–180 HV_10 mN_), so that fan-shaped patterns were also observed in the color-coded contour maps of the hardness distributions, as shown in Figure 16b–d. In the specimen with the highest *O_L_* (i.e., 74.44%), as shown in Figure 16b, the harder cores of the tracks of previous layers were all remelted and covered by the softer boundaries of following layers, so that the fan-shaped pattern is only recognizable in the uppermost layer. On the contrary, fan-shaped patterns with alternating but overall increased hardness values are seen over the layers in the specimens with the lower *O_L_* (i.e., 70.39% and 65.95%), as shown in Figure 16c–d. More of the track cores have been preserved instead of being covered and have undergone a more rapid cooling that is beneficial for hardening. Although the hardness measured at the boundaries is lower than that measured in the cores, it is still higher than that (about 120–130 HV_10 mN_) of the materials in the as-built state, as shown in Figure 16e–g.

### 3.6. Double-Stage Ageing of 3D-Parts

**Figure 17:**
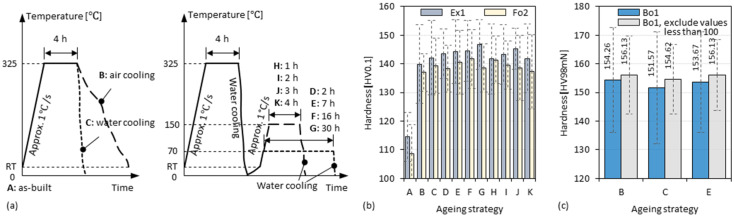
(**a**) Schematic diagram of the single-stage (left) and double-stage (right) ageing strategies; hardness obtained by different ageing strategies in specimen (**b**) Ex1, Fo2, and (**c**) Bo1.

Double-stage ageing strategies were applied to attempt to further enhance the material. Unlike the aforementioned aged specimens which were held at a certain temperature for several hours and then cooled in air, the double-stage aged specimens were, after the first-stage heating, cooled in water and then heated again and held at a lower temperature (i.e., 150 °C or 70 °C) for several hours and finally cooled in water, as shown in Figure 17a. However, no significant further enhancement was found in these specimens compared to the single-stage aged specimens, as shown in Figure 17b,c. Although slightly higher average hardness values (Δ 5–7 HV0.1) were measured in the specimens (i.e., Ex1, Fo2) aged at 70 °C in the second stage, the increase cannot be ruled out as a statistical coincidence, since the measurement values themselves deviate considerably, as shown in Figure 17b. In another specimen (i.e., Bo1), several values that were obviously smaller (i.e., <100 HV_98 mN_) than the average hardness were trimmed to exclude the influence of pores on the statistics. There are still not many differences seen between the specimens, as shown in Figure 17c. This indicates, however, a good tolerance of the material in the exposure to this temperature range.

### 3.7. Tensile Test of 3D-Parts

**Figure 18:**
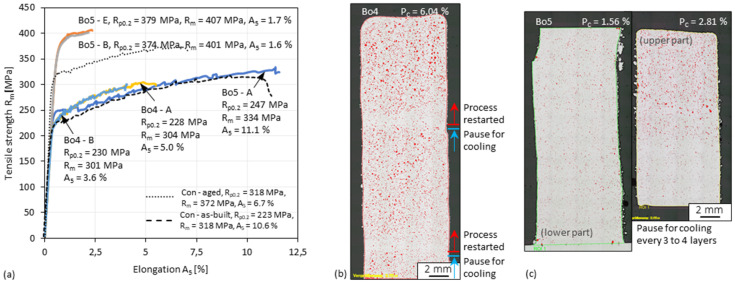
(**a**) Tensile performance of Bo4 and Bo5 in the as-built and aged state. Microscopic images of the cross-section of (**b**) Bo4 and (**c**) Bo5. The specimen “Con-aged (5 h at 300 °C, air cooled)” and “Con-as-built” are reference specimens fabricated by conventional DED using the laser power of 620 W, travel speed of 550 mm/min, powder feed rate of 0.18 g/min, and other settings according to [41] (more details is shown in Figure A2 in Appendix B).

Two specimens (i.e., Bo4 and Bo5) of about ten times the height of the previous ones were prepared for tensile tests. The process parameters used were the same as those for the specimen (i.e., Bo1) with the highest hardness. Considering the repeated heating during the process (i.e., intrinsic heat treatment [53]) and the heat dissipation becomes difficult as the specimen height increases, the two specimens were cooled in two different ways. For Bo4, the process was suspended after the 150th and the 220th layer, respectively, and was restarted after cooling the specimen and the cooling unit to room temperature. The temperature near the top layers of the specimen (i.e., about 3 mm) before cooling was about 300 °C. For Bo5, to ensure the temperature near the top layers did not exceed 120 °C, the process was suspended for cooling the specimen and the cooling unit to room temperature after every 3 to 4 layers. The stress-strain curves of Bo4 in the as-built (i.e., Bo4–A) and aged (i.e., Bo4–B) state are nearly identical and have a tensile strength of about 300 MPa, as shown in Figure 18a. On the contrary, Bo5 has a significantly higher tensile strength increment from 334 MPa to higher than 400 MPa by ageing. As observed in the hardness test, there is little difference between the curves of a single-stage aged (i.e., Bo5–B) and double-stage aged (i.e., Bo5–E) Bo5 sample. A high-level cross-sectional porosity of 6.05% was measured in Bo4, and two zones with fewer pores were observed near where the process was suspended, as shown in Figure 18b. The cross-sectional porosity (i.e., 1.56%) of Bo5 as a whole is generally as low as that of the low porosity zones of Bo4, only slightly higher at the top of the upper part, as shown in Figure 18c, where heat dissipation is less. Although the porosity is still high, the tensile strength of Bo4 after ageing is about 10% higher than that of specimens fabricated by conventional DED, as shown in Figure 18a. Since some specimens (e.g., Ad1 and Bo1) in this work have an average hardness of nearly 150 HV0.1 despite the high porosity, much better tensile performance would be obtained, if the process is applied to alloys (e.g., AlCaScZr [54]) that are less prone to generate pores or if the cooling and inert gas shielding conditions can be further improved.

## 4. Discussion

### 4.1. Process Regimes of DED

**Figure 19:**
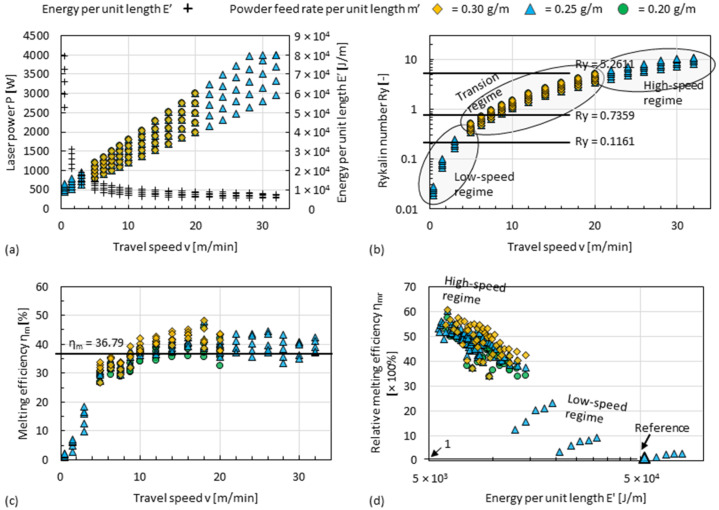
(**a**) Rearranged display of the parameter sets shown in Figure 6a,c and supplementary data of travel speed *v* = 0.5, 1.5 and 3 m/min, and the plot graph of the correspondingly calculated (**b**) Rykalin number *Ry* against travel speed *v*, (**c**) the melting efficiency *η_m_* against the travel speed *v*, and (**d**) relative melting efficiency *η_mr_* against energy per unit length *E’*. Material properties of EN AW-5083 that thermal diffusivity *a* = 5.03 × 10^−5^ W∙m^2^/J, thermal conductivity *k* = 120 W/(m∙K), melting temperature *T_l_* = 638 °C, density ρ = 2650 kg/m^3^ and *c_p_* = 900 J/kg, and of EN AW-5251 that absorption rate *α* = 0.24, and room temperature *T*_0_ = 20 °C are considered for the calculation.

A moving point heat source problem can be treated as a problem in which the heat source stays stationary and the substrate has a backward movement, which causes an advection of heat in the substrate in addition to the heat conduction effect [55]. The Péclet number (Pe) represents the ratio of the heat transport rate by the conduction, and the advection and can be rewritten [55] as a Rykalin number (*Ry*) [56], interpreting the moving source problem:

(3) $$Ry=\frac{v\alpha P}{4\pi ak\left(T_{l}-T_{0}\right)}=\frac{vL}{a}=Pe$$

where *v* is the travel speed, α is the laser absorption rate, *P* is the laser power, *a* is the thermal diffusivity, *k* is the thermal conductivity, *T_l_* is the melting temperature, *T*_0_ is the room temperature, and *L* = *αP*/[4πk(*T_l_* − *T*_0_)] is the characteristic length as related to the temperature gradient at the fusion line induced by the heat source. If the DED process is considered as a simplified problem of a moving point heat source, the combination of the process parameter *v* and *P*, as shown in Figure 19a, determines the *Ry* of the DED process. In moving point heat source problems, an *Ry* of 0.7359 is proposed as the divider between the high-speed regime (more precisely, if *Ry* ≥ 5.2611) and low-speed regime (more precisely, if *Ry* ≤ 0.1161) [55], which are well consistent with the process regimes of DED defined in this work, as shown in Figure 19b.

Melting efficiency *η_m_* characterizes the fraction of energy used to melt the material as a percentage of the total energy input:

(4) $$\eta_{m}=\frac{\rho c_{p}\left(T_{l}-T_{0}\right)vA}{\alpha P}$$

where *ρ* is the density, *c_p_* is the specific heat capacity, and *A* is the cross-sectional area of the fusion zone [57]. In DED, the area *A* can be treated as the sum of the cross-sectional area of the deposition zone A_DE_ and the cross-sectional area of the dilution zone *A_DI_*.

(5) $$A=A_{DE}+A_{DI}$$

In moving point heat source problems, the *η_m_* has a theoretical maximum of 36.79%, since the fusion zone center must be overheated [57]. Taking the measured *A_DE_* and *A_DI_* shown in Figure 8d,e into (4), the *η_m_* of this work can be calculated. It is noted that the practice of DED processes, because of the influence of laser beam shape, powder feeding and the temperature related material properties, etc., deviates from the simplified situation. However, as the travel speed increases, the calculated *η_m_* converges also to an asymptote, and a higher *η_m_* will be achieved, if the powder feed rate per unit length increases, as shown in Figure 19c. Two *η_m_* can be compared using

(6) $$\eta_{mr}=\frac{\eta_{m1}}{\eta_{m2}}=\frac{v_{1}A_{1}P_{2}}{v_{2}A_{2}P_{1}}$$

By taking a value obtained in the low-speed regime as the 100% reference point, the max. *η_m_* obtained in the high-speed regime is about 60 times higher, as shown in Figure 19d.

**Figure 20:**
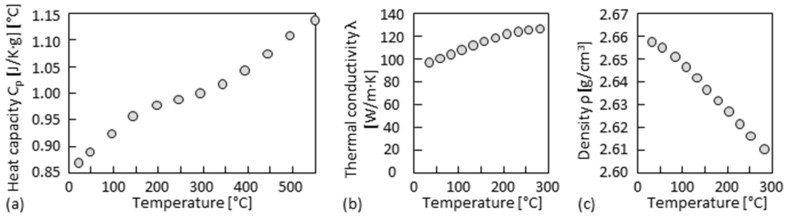
Material property data used in the simulation: (**a**) heat capacity; (**b**) thermal conductivity; (**c**) density.

**Figure 21:**
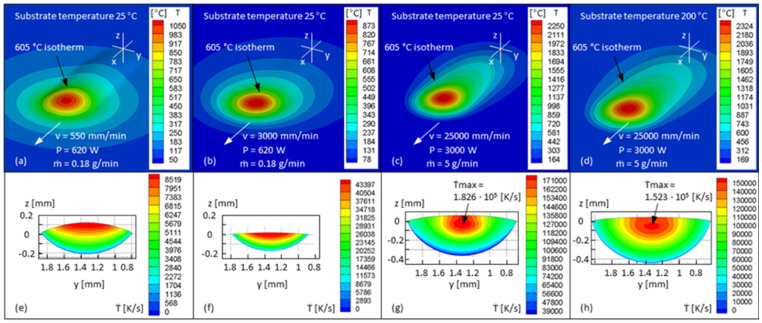
Results of the simulation of the temperature fields (**a**–**d**) and the corresponding cooling rates *Ṫ* on the solidification front of 605 °C isotherm (**e**–**h**) by changing the travel speed *v*, laser power *P*, power feed rate *ṁ*, and the substrate temperature.

More precise heat flow calculations are performed based on a simulation tool developed for DED processes [58,59]. The temperature-dependent heat capacity of the material is measured by DSC (differential scanning calorimetry), as shown in Figure 20a. The thermal conductivity and the density are given by the material supplier, as shown in Figure 20b,c. The solidus (574 °C) and liquidus temperature (638 °C) are adopted from those of 5083 Al-alloy. The heat of fusion (10.79 kJ/mol) and temperature-dependent surface tension (γ(T) = 993 − 0.127T mN/m) are adopted from those of Al. The laser power intensity has a Gaussian distribution. The laser spot diameter is 1.3 mm. Material absorptivity, considering the influence of power conveying, is set at 38%. The heat flow is in quasi-stationary state. The temperature field and the cooling rate on the solidification front of 605 °C isotherm of single tracks are then simulated by changing the travel speed *v*, laser power *P*, power feed rate *ṁ*, and the substrate temperature in the low-speed regime, as shown in Figure 21a,b, and in the high-speed regime (Figure 21c,d). It is seen that the cooling rates at high travel speeds, even with an increased substrate temperature, are still one to two orders of magnitude higher than at low travel speeds.

### 4.2. Ageing Response

**Figure A2:**
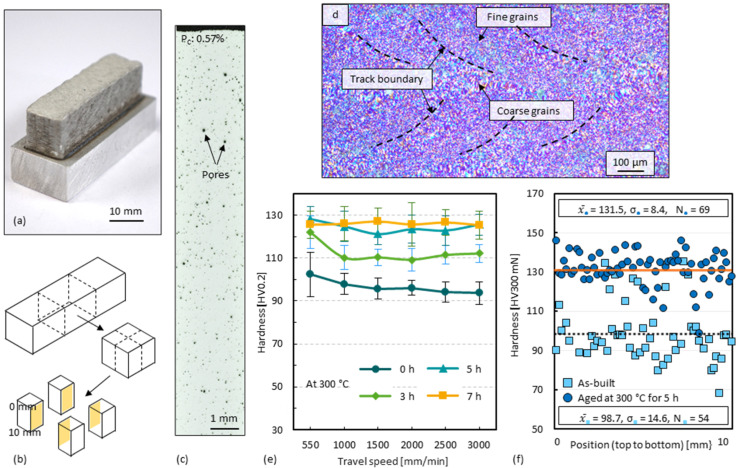
Specimens made of the same material by conventional DED [41], as mentioned in Figure 18 (laser power *P* = 620 W, travel speed *v* = 550~3000 mm/min, powder feed rate *ṁ* = 0.18 g/min, shielding gas flow rate 9 l/min, conveying gas flow rate 5 l/min, laser spot diameter = 1.3 mm, nozzle distance = 7 mm, substrate temperature 18 °C): (**a**) Exterior view of a specimen; (**b**) Sampling position for metallographic analyses; (**c**) cross-section of the specimen and measured cross-sectional porosity *P_C_* of 0.57%; (**d**) Barker-etched cross-section; (**e**) Average micro hardness values of samples in different aging states (30 indentations from the top to the bottom of each sample; error bars indicate one standard deviation); (**f**) Values of hardness tests using much lower loads from the top to the bottom (abnormal hardness values less than 0.8 and more than 1.2 times of the mean value of the hardness of five adjacent indentations were filtered out as the indentations on pores. $\bar{x}$: average hardness value; *σ*: standard deviation; N: number of data points; dashed line: average hardness of as-built specimens; solid line: average hardness of aged specimens).

The inadequate ageing response of specimens of AlMgScZr alloys fabricated with DED is considered as an important reason for their poorer mechanical properties compared to the process outcome of LPBF. The as-built specimens fabricated with conventional DED exhibit a hardness level of about 100 HV0.1, for example, the 102 HV0.1 of the specimen fabricated with 550 mm/min as shown in Figure A2e, which is close to that (e.g., 105 HV0.3 [28]) of the as-built specimens fabricated with LPBF. However, the hardness of the specimen fabricated with conventional DED was only increased by 26 HV0.2 (i.e., 255 MPa in SI unit) by ageing at 300 °C for 5 h. The yield strength of the specimen fabricated and aged in the same way was recorded with a yield strength increase of 95 MPa, as shown in Figure 18a. Such an ageing response is only about 40% as high as that obtained in specimens fabricated with LPBF, for example, an increment of 72 HV0.3 in hardness [28] and 234 MPa in yield strength [49]. The proportional relationship between this hardness increment and the yield strength increment observed in conventional DED and LPBF roughly corresponds to the empirical Tabor’s relationship [60]:

(7) $$H\approx 3\cdot \sigma_{y}$$

where *H* is the hardness in SI unit and *σ_y_* is the uniaxial yield strength. The highest hardness increment observed in this work in the aged specimens fabricated with high-speed DED was 47 HV0.1 (i.e., 460.6 MPa in SI unit), as shown in Figure 13a, which according to the Tabor’s relationship should correspond to a yield strength increment of about 153 MPa. The actual measured yield strength increment of 132 MPa (i.e., Bo5–E vs. Bo5–A, as shown in Figure 18a), although slightly lower than this value, is despite the higher porosity level about 40% higher than that observed in conventional DED and is further close to the outcome of LPBF.

## 5. Conclusions

This work demonstrates laser directed energy deposition (DED) of AlMgScZr alloys in high-speed process regimes and elucidates the mechanism of enhancing the hardness and tensile strength of AlMgScZr alloys by increasing the cooling rate during the process as well as reducing the track overlapping and the porosity of the specimen. The results of this study indicate that:

1. Cooling rates of Al alloys in high-speed regimes (travel speed ≥20 m/min) can theoretically reach up to about 2.34 × 10^5^ K/s. This is an increase of 1–2 orders of magnitude compared to that of conventional DED, thus increasing the overall precipitation strengthening potential of every single track.
2. Since single tracks of AlMgScZr alloys are observed to have soft fringes and hard cores on their cross-sections, decreasing the amount of remelted area when tracks overlap can increase the percentage of the hard zones. This can be achieved by decreasing the energy per unit length *E’* (= Laser power *P*/travel speed *v*) and increasing the powder feed rate per unit length *m’* (= powder feed rate *ṁ*/travel speed *v*), ultimately obtaining a high deposition-to-dilution ratio of 30%, which allows one to fabricate bulk 3D-parts having an average hardness of nearly 150 HV0.1 and a tensile strength of 407 MPa (with *E’* = 5400 J/m, *m’* = 0.25 g/m and ageing at 325 °C for 4 h).
3. Concepts that have better cooling and Ar shielding conditions are proven effective, and these are beneficial for reducing the porosity and improving the mechanical performance of the material. Notwithstanding that the current experimental setup does not allow for more precise studies regarding the material oxidation during the high-speed DED process, the signs of trends observed in this work suggest there would be a fruitful area for further work.

## Figures and Tables

**Table 1:** Parameter sets of laser power *P*, travel speed *v*, nozzle’s own shielding gas flow rate *V_s_*, additional shielding gas flow rate *V_ad_*. and shielding and exhausting measures for fabricating 3D-parts. (Laser spot Ø = 1.3 mm. Conveying gas flow rate = 5 L/min. Powder feed rate = 5 g/min.).

Sample	Shielding and Exhausting Measure	*P* [W]	*v* [m/min]	*V_s_* [L/min]	*V_ad_* [L/min]
Or1	Nozzle’s original shielding	2500	20	10	-
Ad1	With an additional shielding gas flow	1800	20	20	10
Ad2		2500	20	20	10
Ad3		2500	25	20	10
Ad4		3000	25	20	10
Ad5		3500	25	20	10
Ri1	With a shielding sleeve	2500	20	10	-
Ri2		2500	20	10	-
Ex1	With an active smoke exhaust flue	1800	20	10	-
Ex2		2500	20	10	-
Ex3		2500	20	10	-
Ex4		2500	20	10	-
Ex5		2500	20	10	-
RE1	With a shielding sleeve	2500	20	10	-
	and an active smoke				
	Exhaust flue				
Fo1	Covered with an Al curtain filled with Ar	1800	20	10	-
Fo2		2500	20	10	-
Fo3		1800	20	10	-
Fo4		2500	20	20	-
Bo1	Covered with a protective box filled with Ar	1800	20	10	-
Bo2		2200	20	10	-
Bo3		2500	20	10	-
Bo4		1800	20	10	-
Bo5		1800	20	10	-

**Table 2:** Heat treatment strategies for AlMgSc(Zr) alloys fabricated by different processes and the achieved mechanical properties.

Material	Process	Optimum Heat Treatment	Best Mechanical Properties Achieved	Source
Al–6Mg–0.2Sc	Casting	1 h at 300 °C	Hardness: 69 HV5	[48]
Al–6Mg–0.4Sc			Hardness: 76 HV5	
Al–6Mg–0.6Sc			Hardness: 81 HV5	
Al–4Mg–0.4Sc–0.12Zr	Casting	3 h at 325 °C	Hardness: 95 HV0.1	[22]
	& electron beam		Hardness: 110 HV0.1	
Al–4.5Mg–0.66Sc–0.37Zr	LPBF	4 h at 325 °C	Hardness: 177 HV0.3	[28]
			UTS: 530 MPa, A: 14 %	
Al–4.6Mg–0.66Sc–0.42Zr	LPBF	4 h at 400 °C	Hardness: 137 HB	[30]
		5 h at 325 °C	Hardness: 145 HB	
		4 h at 325 °C – 350 °C	UTS: 515 MPa (vertically built)	
			UTS: 530 MPa (horizontally built)	
Al–2.9Mg–1.11Sc–0.42Zr	LPBF	1 h at 540 °C, water quenching,	UTS = 541.3 MPa, A: 0.7%	[49]
		then 2 h at 325 °C		
Al–4.2Mg–0.4Sc–0.2Zr	DED	5 h at 300 °C	Hardness: 129 HV0.2	[41]
			UTS: 347 MPa, A: 5.1%	
Al–4.82Mg–0.25Sc–0.12Zr	DED	4 h at 325 °C	Hardness: 97.9 HV0.2	[40]
Al–4.87Mg–0.35Sc–0.16Zr			Hardness: 100 HV0.2	
Al–4.86Mg–0.50Sc–0.21Zr			Hardness: 105 HV0.2	

**Figure A1:**
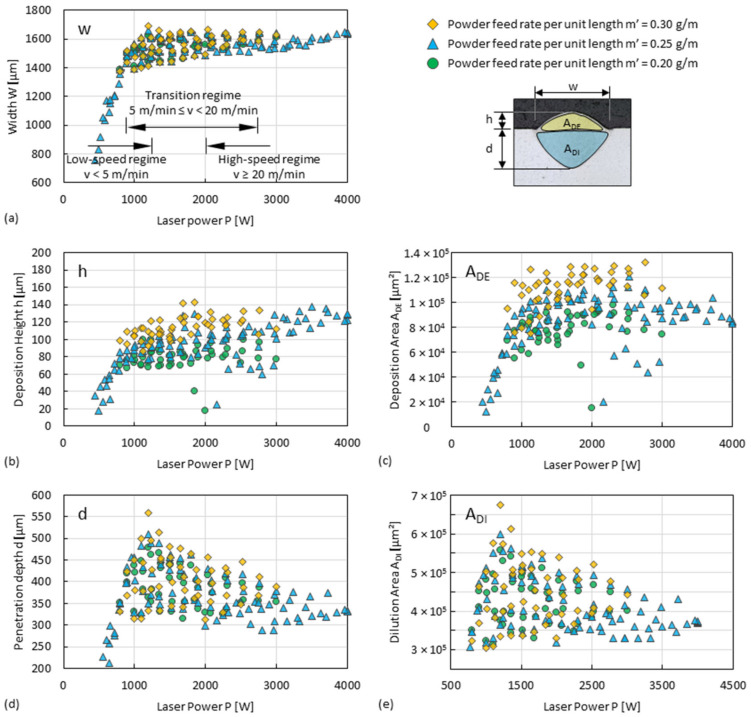
Plot graphs of the data shown in Figure 8 and supplementary data of “low-speed regime” (also see Figure 19) of (**a**) track width *w*, (**b**) track height *h*, (**c**) cross-sectional area of the deposition zone *A_DE_*, (**d**) melt pool depth *d*, and (**e**) cross-sectional area of the dilution zone A_DI_ against laser power *P* with different powder feed rate per unit length *m’*. Every point refers to the average value of three measurements. The corresponding travel speeds are marked in (**a**).

## Data Availability

Not applicable.
